# Obstetrics: The Science and the Art

**Published:** 1852-07

**Authors:** 


*Obstetrics: the Science and the Art.* By Charles D. Meigs, M. D., Professor of Midwifery and Diseases of Women and Children in Jefferson Medical College, at Philadelphia, &c. &c. Second edition, revised. Philadelphia, Blanchard & Lea, 1852.

Professor Meigs has long since taken the first rank among original writers on American medicine. His works on obstetrics and kindred subjects have rapidly gone through frequent editions, and have been well received both abroad and at home. There are about them a freshness, vivacity, and energy of style and thought, which, though sometimes degenerating into oddities and whimsicalities, have certainly proved attractive. Some of the critics have, indeed, carped at his manner, as wanting in the severity and dignity which should belong to the subject; but we suppose these rapidly succeeding editions of his books are a sufficient evidence that he has pitched his key to suit the ears of those for whom he writes.

The "Obstetrics" is an enlargement of the "Philadelphia Practice of Midwifery," which had previously gone through two editions. The first edition of the present work was published in 1849. "Though a large one, it was soon exhausted;" and of the edition before us, just issued, the author states that he has "considerably augmented it as to the text, endeavored to improve it by recasting some parts, cancelling others, and by an earnest attention to improvements in the literary execution of the whole." Our opinion of the first edition having been already expressed at some length, we must content ourselves here with briefly commending the book to the American student, as one of the best manuals on the subject extant, and adapted to his requirements. It were superfluous to say, that there is no better living teacher of the physiology, mechanism, therapeutics and surgery of obstetrics, than Dr. Meigs; and his book contains all that is known on these points, most graphically described, and aptly illustrated from the records of an experience, we presume now second to none in the world.

That Dr. Meigs entertains peculiar or rather extreme views on the pathology of some diseases of women and children, is well known to all who are conversant with the medical literature of the day. These views have been reiterated through most of his books; they have been discussed at some length in our columns; and we feel called upon here merely to say that we remain among those who cannot subscribe to all his doctrines "upon the nature, seats, causes and treatment of puerperal or child-bed fever," particularly as to its non-contagiousness.

